# Hormonal Regulation in Shade Avoidance

**DOI:** 10.3389/fpls.2017.01527

**Published:** 2017-09-04

**Authors:** Chuanwei Yang, Lin Li

**Affiliations:** State Key Laboratory of Genetic Engineering, Institute of Plant Biology, School of Life Sciences, Fudan University Shanghai, China

**Keywords:** shade avoidance syndrome, light signaling, PIFs, hormone regulation, crosstalk

## Abstract

At high vegetation density, shade-intolerant plants sense a reduction in the red (660 nm) to far-red (730 nm) light ratio (R/FR) in addition to a general reduction in light intensity. These light signals trigger a spectrum of morphological changes manifested by growth of stem-like tissue (hypocotyl, petiole, etc.) instead of harvestable organs (leaves, fruits, seeds, etc.)—namely, shade avoidance syndrome (SAS). Common phenotypical changes related to SAS are changes in leaf hyponasty, an increase in hypocotyl and internode elongation and extended petioles. Prolonged shade exposure leads to early flowering, less branching, increased susceptibility to insect herbivory, and decreased seed yield. Thus, shade avoidance significantly impacts on agronomic traits. Many genetic and molecular studies have revealed that phytochromes, cryptochromes and UVR8 (UV-B photoreceptor protein) monitor the changes in light intensity under shade and regulate the stability or activity of phytochrome-interacting factors (PIFs). PIF-governed modulation of the expression of auxin biosynthesis, transporter and signaling genes is the major driver for shade-induced hypocotyl elongation. Besides auxin, gibberellins, brassinosteroids, and ethylene are also required for shade-induced hypocotyl or petiole elongation growth. In leaves, accumulated auxin stimulates cytokinin oxidase expression to break down cytokinins and inhibit leaf growth. In the young buds, shade light promotes the accumulation of abscisic acid to repress branching. Shade light also represses jasmonate- and salicylic acid-induced defense responses to balance resource allocation between growth and defense. Here we will summarize recent findings relating to such hormonal regulation in SAS in *Arabidopsis thaliana*, *Brassica rapa*, and certain crops.

## Introduction

Over the past few decades, a substantial body of studies has focused on understanding how plants sense the proximity of neighbors, how they respond at molecular levels, and how they adjust their morphological and physiological indexes. Many important light signaling components have been shown to regulate the shade avoidance responses—for example, PIFs (phytochrome interacting factors), HFR1 (long hypocotyl in far-red 1), PAR1/2 (phytochrome rapidly regulated 1/2) and COP1 (constitutive photomorphogenic 1). Meanwhile, various phytohormones are also involved and coordinated to shape shade-regulated plant architecture. Analyses of hormonal biosynthetic and signaling mutants, combined with studies of exogenous hormone applications, have implicated the roles of these phytohormones in multiple shade avoidance responses. In this review, we provide an overview of the current understanding of shade light and subsequent hormonal regulation.

## Shade Signal and Plant Perception

Light-quality signals are of paramount importance in detecting neighboring vegetation. Photosynthetic pigments in leaves absorb strongly in the range of photosynthetically active radiation (PAR) (400–700 nm) and UV radiation (280–400 nm), and reflect far-red wavelength (700–800 nm) (Casal, 2013). Thus, natural shade is a combination of the reduction in the red/far-red ratio (R/FR), the reduction in red plus far-red irradiance, the reduction in blue and UV irradiance, and the reduced blue/green ratio. To detect these spectral differences, plants use multiple light sensors, such as red and far-red light absorbing phytochromes, the blue/UV-A light sensing cryptochromes, and the UV-B photoreceptor protein (UVR8).

## A Brief Account of the Shade Signaling Pathway

Phytochromes exist in two photoconvertible forms: an inactive R-absorbing Pr form and an active FR-absorbing Pfr form. The steady-state ratio of Pr and Pfr forms depends on R/FR. The constitutive shade avoidance syndrome (SAS) phenotype of *Arabidopsis phyB* mutant plants indicates that phyB plays a dominant role in inhibiting SAS (Franklin and Quail, 2010). High R/FR establishes a high proportion of phyB Pfr, which interacts with the bHLH family of transcription factor PIFs and triggers the phosphorylation, ubiquitination and degradation of PIFs. In contrast, low R/FR drives Pfr-to-Pr conversion and releases the suppression of PIFs. Activated PIFs promote gene expression related to shade-induced growth. PIF7, PIF4 and PIF5 play central roles in this process (Lorrain et al., 2008; Li L. et al., 2012).

To prevent exaggerated shade-avoidance responses, shade-induced HFR1 (Sessa et al., 2005; Hornitschek et al., 2009), PAR1/2 (Roig-Villanova et al., 2006; Galstyan et al., 2011; Bou-Torrent et al., 2014), and PIL1 (PIF3 like 1) (Li et al., 2014; Luo et al., 2014) are proposed as the negative regulators of PIFs. The bZIP transcription factor, elongated hypocotyl 5 (HY5), is also reported to form non-functional complexes with PIFs (Chen et al., 2013; Toledo-Ortiz et al., 2014). In addition to directly binding with PIFs, the Suppressor of phyA-105 (SPA)/COP1 E3 ubiquitin ligase complex indirectly enhances PIF activity by degrading HFR1 and HY5 to augment shade responses (Sheerin et al., 2015; Pacin et al., 2016). BBX (double B-box) 21 and BBX25 regulate shade response through the function in the COP1 signaling pathway (Crocco et al., 2010; Gangappa et al., 2013).

Cryptochromes (CRYs) are involved in repressing a low blue-mediated SAS by regulating PIF abundance and activity (de Wit et al., 2016; Pedmale et al., 2016). PIF activity is enhanced directly through CRY inactivation and indirectly through relieved inhibition of COP1, which increases the degradation of negative regulators of PIF, including HFR1 and HY5 (de Wit et al., 2016).

UV-B-mediated inhibition of shade responses has been reported to occur through the degradation of PIF4/5 (Hayes et al., 2014).

In summary, downstream of photoreceptors, PIFs, as the key regulators, determine the massive transcriptional reprogramming upon perception of shade light, and also mediates the convergence between light and hormones.

## Auxin, A Prominent Player in Shade-Induced Elongation Growth

A forward genetic screen for impaired shade-induced hypocotyl elongation in *Arabidopsis* identified TAA1, an enzyme catalyzing the first step of an auxin biosynthetic pathway (Tao et al., 2008; Won et al., 2011). Later, a family of enzymes encoded by *YUCCA* (*YUC*) genes has been functionally positioned as the second and rate-limiting step of TAA1-dependent auxin biosynthesis (the indole-2-pyruvic acid pathway, or “IPA pathway”). The transcriptional regulation of *YUCCA* genes by PIF7 has been found to link photoperception with auxin biosynthesis (Li L. et al., 2012). The level of shade-stimulated free indole-3-acetic acid (IAA) is blunted in *taa1*, and *pif7* mutants confirm that auxin production through the TAA1-YUC pathway is required to initiate the SAS in seedlings (Tao et al., 2008; Li L. et al., 2012; Procko et al., 2014). PIF4 and PIF5 are partially redundant, with PIF7 regulating the expression of *YUCCA* genes (Hornitschek et al., 2012). Correspondingly, the *yuc2 yuc5 yuc8 yuc9* quadruple mutant displays the completely disrupted SAS (Nozue et al., 2015; Muller-Moule et al., 2016). Tissue-level measurement in *Brassica rapa* seedlings has suggested that auxin appears to be generated in the cotyledons and transported to the hypocotyl (Procko et al., 2014). Indeed, seedlings treated with the auxin transport inhibitor naphthylphalamic acid (NPA) totally abolish shade-induced hypocotyl elongation (Tao et al., 2008). Consistently, *pin3-3* (PIN3, auxin transporter) exhibits an impaired shade-induced hypocotyl elongation (Keuskamp et al., 2010), and the mutation in *SAV4* leads to defective basipetal auxin transport and shade responses (Ge et al., 2017), indicating that auxin redistribution is important for shade-avoidance reactions (Morelli and Ruberti, 2000).

Besides auxin biosynthesis and transport, auxin sensitivity is also enhanced under shade (Nozue et al., 2011; Hornitschek et al., 2012; Bou-Torrent et al., 2014). Auxin signaling components, such as AUX/IAAs (Auxin/indole-3-acetic acid), have been reported to modulate the SAS (Steindler et al., 1999; Procko et al., 2016).

In addition to *Arabidopsis*, the key role of auxin on the SAS has also been confirmed in crop species (Carriedo et al., 2016). Shade-induced changes in auxin level have been found in sunflower (Kurepin et al., 2007) and tomato (Kozuka et al., 2010). Expression quantitative trait locus (eQTL) analysis identified a group of auxin-related genes, which were down-regulated in shade-tolerant tomato lines and up-regulated in the shade responders, suggesting the role of auxin in the natural variation of the SAS (Bush et al., unpublished). In maize seedlings (Wang et al., 2016) and rice seedlings (Liu et al., 2016), the expression of auxin-responsive genes is also dramatically affected by shade treatment.

Considered together, it may be concluded that intact auxin biosynthesis, transportation and signaling are required for shade-induced stem growth.

## Gibberellin, Another Shade Growth-Promoting Hormone

Shade treatment resulted in an increased gibberellin (GA) concentration in bean internode (Beall et al., 1996), cowpea (*Vigna sinensis*) epicotyls (Martínez-García et al., 2000), sunflower stem (Kurepin et al., 2007) and *Arabidopsis* seedling (Bou-Torrent et al., 2014). The shade-induced GA biosynthetic enzymes *GA20ox1*, *GA20ox2*, and *GA3ox* at least in part account for the increase in active GA (Hisamatsu et al., 2005; Yu et al., 2015).

Bioactive GA leads to proteasomal degradation of DELLA proteins (Harberd et al., 2009). Lacking direct DNA binding capability, DELLAs are direct interactors of PIFs. Their binding prevents PIF proteins from binding DNA and thus negatively regulates the expression of genes involved in cell elongation (de Lucas et al., 2008; Feng et al., 2008). Shade-induced breakdown of DELLA proteins due to increased gibberellin biosynthesis releases the suppression of PIFs, and activates the transcription of target genes. The GA-insensitive *gai* gain-of-function mutant, which has a stable GAI (DELLA) protein, shows a reduced SAS (Djakovic-Petrovic et al., 2007), suggesting that DELLA proteins constrain the SAS.

It is noteworthy that proteins that physically interact with DELLA proteins may alleviate DELLA-mediated repression of PIF activity, such as BBX24. The shade-response defect in *bbx24* mutants is rescued by a GA treatment (Crocco et al., 2015).

In addition to GA-induced seedling phenotypes, GA biosynthesis and signaling are also important for shade-induced flowering. Silencing *GA20ox2* expression delays flowering of *Arabidopsis* exposed to a FR-enriched light condition (Hisamatsu and King, 2008).

## Ethylene, An Organ-Specific Regulator of the SAS

Low R/FR can enhance the production of ethylene in wide-type tobacco (Pierik et al., 2004). In *Arabidopsis*, shade-induced petiole elongation was absent in the ethylene-insensitive mutants *ein2-1* and *ein3-1eil1-3*, indicating that ethylene is a positive regulator of shade-induced petiole elongation (Pierik et al., 2009). However, the *ein3eil1* mutant retains a full shade-induced hypocotyl response (Das et al., 2016). The controversy suggests that ethylene plays a role in organ-specific shade response.

A recent research shows that light activation of photoreceptor phyB results in rapid degradation of EIN3, a master transcription factor in the ethylene signaling pathway (Shi et al., 2016). The position of ethylene signaling components under shade is worthy of further investigation.

## Brassinosteroid, A Dynamic Regulator Under Shade

The promotion of stem growth by shade light requires brassinosteroids (BRs) because the BR biosynthesis mutant *dwarf1* (Luccioni et al., 2002) and *rot3* (Kim et al., 1998) are unable to show the elongation of hypocotyl under shade, as with wild-type seedlings treated with the BR synthesis inhibitor brassinazole (Keuskamp et al., 2011). BR biosynthesis is also required for petiole growth under low R/FR (Kozuka et al., 2010). However, short-term (4 h) simulated shade treatments resulted in lower levels of the active BR, and longer periods (24 h) abolished the differences in BR levels in whole seedlings (Bou-Torrent et al., 2014), suggesting that simulated shade altered BR levels in a dynamic fashion.

Beside the level of hormones, the sensitivity of seedlings to hormones also has an important effect on shade-induced growth. BR signaling components BR-ENHANCED EXPRESSION (BEE) and BES1-INTERACTING MYC-LIKE (BIM) are positive regulators of SAS hypocotyl responses because *bee123* and *bim123* seedlings display hypocotyl elongation defects after detecting simulated shade (Cifuentes-Esquivel et al., 2013). Remarkably, DELLAs negatively regulate BR signaling by binding BZR1 and reducing the expression of BR-responsive genes (Bai et al., 2012; Gallego-Bartolome et al., 2012; Li Q.F. et al., 2012). The transcription factor BZR1 and PIF4 physically interact and synergistically regulate target genes (Oh et al., 2012; Kohnen et al., 2016). Given that the binding of DELLA and PIFs impair the DNA-binding ability of PIFs, the complex of DELLAs, BZR1, and PIFs may play a role in stem elongation, and possibly exerts a similar function in shade avoidance, but this needs further investigation (Casal, 2013; de Lucas and Prat, 2014). In concordance with these findings, BR-responsive genes are overrepresented in end-of-day FR-induced genes in both the leaf blade and petiole (Kozuka et al., 2010). Although the majority of the BR genomic response comprises genes annotated as auxin responsive, the regulation of BR and auxin on SAS responses might nevertheless occur in a non-redundant and non-synergistic manner, because the response to blue light depletion will be fully inhibited only when both hormones are blocked simultaneously (Keuskamp et al., 2011).

In particular, the BR response appears to be required for the full expression of the SAS phenotypes under low blue light (Keller et al., 2011; Keuskamp et al., 2011). The question as to how BR biosynthesis and signaling dynamically respond to low R/FR or low blue light is yet to be answered.

## Cytokinin, Ensuring Reallocation of Plant Resources

The role of cytokinins (CKs) in shade avoidance responses was discovered from the response of plants to vertical light intensity gradients in leaf canopies (Pons et al., 2001). In shaded leaves, where stomatal conductance and transpiration rate are reduced, the low delivery rate of CKs leads to reduced photosynthetic capacity and ultimately senescence (Boonman and Pons, 2007).

Another role of CKs was found in the inhibition of leaf growth in shade. Low R/FR signal can induce hypocotyl elongation and also trigger a rapid arrest of leaf-primordia growth by the breakdown of auxin-induced CKs through the action of *AtCKX6* (cytokinin oxidase) in the incipient vein cells of developing primordia (Carabelli et al., 2007). In addition, the CK receptor AHK3 has been reported to mediate the root-to-hypocotyl ratio response under shade conditions (Novak et al., 2015).

The reduction of bioactive CKs triggers a reduced photosynthetic capacity and a transient arrest of leaf development, ensuring that energy resources are indeed redirected into extension growth in shade.

## Jasmonic Acid, Shade-Reduced Hormone Related to Defense

Plants often display a weak defense in insect and pathogen infection under shade conditions or FR-enriched conditions (Cerrudo et al., 2012; de Wit et al., 2013; Ballare, 2014). Shade has been shown to reduce herbivory-induced jasmonic acid (JA) accumulation (Agrawal et al., 2012), and FR-exposed plants suffer more insect herbivory than wild-type plants (Moreno et al., 2009), suggesting that shade can down-regulate the JA pathway to control plant immunity.

The JAZ-DELLA pathway is an important modulator of plant immunity under shade conditions (Moreno and Ballare, 2014). DELLA proteins positively regulate JA signaling by interacting with JAZs, and this interaction weakens the ability of JAZs to repress MYC2 (Hou et al., 2010; Yang et al., 2012). As described previously, DELLA proteins negatively regulate growth-related genes by binding PIFs (de Lucas et al., 2008; Feng et al., 2008). JAZ10 is required for the inhibitory effect of shade on JA responses (Leone et al., 2014). Therefore, shade conditions induce GA synthesis and the degradation of DELLA proteins, consequently increasing PIF-dependent growth and impairing JAZ-dependent defense. Canopy shade represses JA-mediated defenses via shade-induced stabilization of JAZ proteins and triggers inactivation of MYC2, MYC3, and MYC4 proteins (Chico et al., 2014). By contrast, regulation of the protein stability of MYCs and JAZs by shade facilitates reallocation of resources from defense to growth. The mutants deficient in JA biosynthesis and signaling display exaggerated shade-induced hypocotyl responses to a low R/FR ratio (Robson et al., 2010). Moreover, several FR light induced gene expressions are dependent on CORONATINE INSENSITIVE1 (COI1), a central component of JA signaling (Robson et al., 2010).

Canopy light cues affect emission of constitutive and methyl JA-induced volatile organic compounds, which can be detected by herbivorous insects (Kegge et al., 2013). A recent study found that in tomato (*Solanum lycopersicum*) phyB inactivation led the plants to produce a blend of JA-induced monoterpenes that increased their attractiveness to the predatory mirid bug *Macrolophus pygmaeus* (Cortes et al., 2016; Ballare and Pierik, 2017).

Certain transcription factors in the JA signaling pathway also participate in the regulation of SAS; for example, PHYTOCHROME AND FLOWERING TIME 1 (PFT1), a subunit of Mediator, is required for both JA-dependent defense gene expression and shade-induced early flowering (Cerdan and Chory, 2003; Cevik et al., 2012; Inigo et al., 2012). These factors could be the additional linkers of light signal and JA-mediated defenses.

## Salicylic Acid, Another Shade-Reduced Hormone

Salicylic acid (SA)-dependent disease resistance is also reduced under shade, which is considered as the early warning signal for plant competition (de Wit et al., 2013). Reduced SA synthesis (Griebel and Zeier, 2008) and response (de Wit et al., 2013) have been correlated with phyB inactivation. Under a low R/FR ratio, the phosphorylation level of the SA-signaling component NONEXPRESSOR of PATHOGENESIS-RELATED GENE 1 (NPR1) is reduced, which partly explains why shade reduces SA-dependent disease resistance. A more detailed explanation of the mechanism that exists between shade avoidance responses and SA is required.

## Abscisic Acid, Repressing Branching Under Shade

Abscisic acid (ABA) is commonly known as the “stress hormone” that responds to a variety of environmental stresses including both biotic and abiotic stress. Shade conditions increase ABA levels in sunflower (*Helianthus annuus*) (Kurepin et al., 2007) and tomato leaves (Cagnola et al., 2012). Shade increases the endogenous ABA level probably by enhancing the transcript levels of ABA biosynthetic gene *NINE-CIS-EPOXYCAROTENOID DIOXYGENASE 3* (*NCED*3) and *NCED5*, particularly in hypocotyls (Kohnen et al., 2016). Several ABA signaling genes (*ABF3*, *AFP1*, *AFP3*, and *GBF3*) are up-regulated by a neighbor signal (Sellaro et al., 2017).

Shade light exerts a strong influence on branch development (Finlayson et al., 2010; Su et al., 2011). One recent study suggested that shade represses branching in bud n-2 by accumulation of ABA (Reddy et al., 2013). The genes involved in ABA biosynthesis and signal transduction showed varied gene expression patterns in responsive buds with increasing R/FR treatment. ABA biosynthesis mutants (*nced3-2* and *aba2-1*) exhibited enhanced branching capacity under low R/FR.

However, ABA was not involved in shade-induced petiole elongation (Pierik et al., 2011), suggesting that the roles of ABA in the SAS may be organ specific.

## Strigolactone, An Unclear Role in the SAS

Most shade-avoiding plants display reduced branching and enhanced apical growth, which helps them to compete for incident light. Strigolactone (SL) is one of the hormones that control lateral shoot growth. In *Arabidopsis*, *BRC1* (*BRANCHED1*) is up-regulated in the axillary buds of plants grown at high density and is required for shade-mediated branch suppression (Aguilar-Martinez et al., 2007; Gonzalez-Grandio et al., 2013). In sorghum, inhibition of outgrowth in a *phyB* mutant and by FR treatment is correlated with an increase in the transcript levels of the SL-signaling gene *SbMAX2* in buds (Kebrom et al., 2010). The involvement of SL in SAS has been observed, but more detailed studies of this mechanism are required.

Besides branching, *Arabidopsis max2* mutants show longer hypocotyls under red, far-red and blue light than wild–type plants (Shen et al., 2012; Jia et al., 2014). The double mutant *pif1max2* shows a similar hypocotyl length to *max2*, which indicates that *MAX2* is epistatic to *PIF1* (Shen et al., 2012). *MAX2* plays a role in the light signaling pathway, but further investigation of the mechanisms involved is needed.

## Karrikins, A Possible Way to Attenuate the SAS

Studies have shown that karrikins enhance the sensitivity of seedlings to light (Waters and Smith, 2013). Since karrikins can inhibit elongation of the hypocotyl and increase the chlorophyll content (Nelson et al., 2010), they may be an efficient solution to attenuating plant SAS during the seedling stage (Meng et al., 2016).

## Final Remarks

**FIGURE 1:**
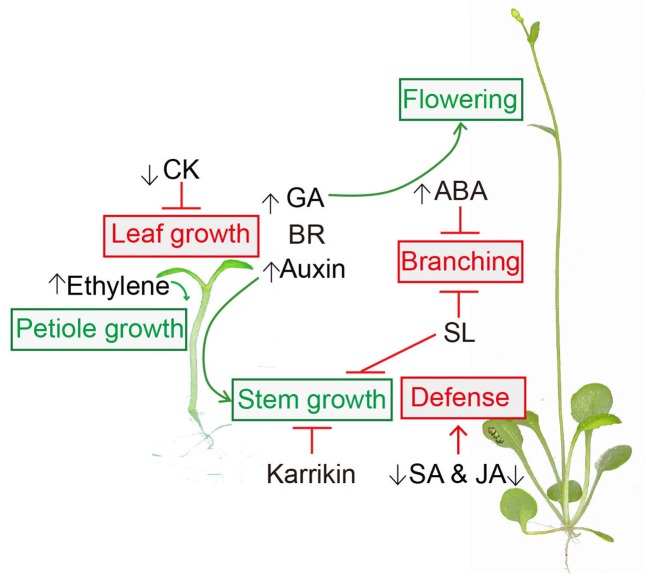
Hormonal regulation in shade avoidance. Auxin, Gibberellin (GA), Brassinosteroid (BR), Karrikin and strigolactone (SL) are involved in shade-regulated stem growth. Ethylene is required for shade-induced petiole elongation. Shade-reduced cytokinin (CK) inhibits the leaf growth. Shade light also represses salicylic acid (SA) and jasmonic acid (JA) mediated defense. Abscisic acid (ABA) and SL suppress branching in shade. GA contributes to shade-induced early flowering. Shade-stimulations are presented in green and shade-supressions are presented in red.

This review focused on understanding the interaction between phytohormones and the SAS (**Figure 1**). The regulations of these phytohormones on the SAS described here might vary according to tissue type (Kohnen et al., 2016), stage of development (Roig-Villanova and Martinez-Garcia, 2016) and species (Liu et al., 2016). In this regard, further research into the spatial and temporal regulation of phytohormones is necessary for a mechanistical understanding of the SAS. Moreover, crosstalk among hormones under shade conditions is also worthy of further investigation.

## Author Contributions

CY and LL designed and wrote the manuscript.

## Conflict of Interest Statement

The authors declare that the research was conducted in the absence of any commercial or financial relationships that could be construed as a potential conflict of interest.
